# Hierarchy of Chaotic Dynamics in Random Modular Networks

**DOI:** 10.1103/PhysRevLett.134.148402

**Published:** 2025-04-11

**Authors:** Łukasz Kuśmierz, Ulises Pereira-Obilinovic, Zhixin Lu, Dana Mastrovito, Stefan Mihalas

**Affiliations:** Allen Institute, Seattle, Washington, USA

## Abstract

We introduce a model of randomly connected neural populations and study its dynamics by means of the dynamical mean-field theory and simulations. Our analysis uncovers a rich phase diagram, featuring high- and low-dimensional chaotic phases, separated by a crossover region characterized by low values of the maximal Lyapunov exponent and participation ratio dimension, but with high values of the Lyapunov dimension that change significantly across the region. Counterintuitively, chaos can be attenuated by either adding noise to strongly modular connectivity or by introducing modularity into random connectivity. Extending the model to include a multilevel, hierarchical connectivity reveals that a loose balance between activities across levels drives the system towards the edge of chaos.

## Introduction—

Natural and artificial systems at the edge of chaos exhibit unique computational properties [1-4]. For instance, placing artificial neural networks at the edge of chaos has been shown to optimize learning and information processing in various scenarios [5-10]. Accordingly, it has been suggested that neural circuits in the brain operate at, or remain near to, a critical point such as the edge of chaos [11-19], which may explain some aspects of the structure of the variability observed in neural recordings. However, keeping complex systems at a critical point is challenging, as it is usually sensitive to external or internal changes, such as noise and external inputs [10,20-22], or low-rank structural connectivity perturbations [23-30]. How can complex systems be robustly maintained at the edge of chaos? In the context of neuroscience, synaptic plasticity has been suggested as the underlying feedback mechanism driving neural circuits towards a critical point [31-34]. It is unclear, however, whether synaptic plasticity mechanisms can drive networks specifically to the edge of chaos [35] and how the underlying connectivity organization affects the type and robustness of the emergent critical behavior.

Hierarchical organization is common in biological and artificial complex systems, including brains, ecosystems, and artificial neural networks. Mammalian brains are modular and hierarchical [36-41], and are composed of many distinct cell types that themselves are hierarchically organized [42,43]. The hierarchical organization of the visual system [44] has inspired the design of computationally powerful artificial neural network architectures [45], which are similarly hierarchically structured [46]. Ecological systems, including food webs, also exhibit hierarchical organization [47]. How does hierarchical organization affect the collective behavior of such complex systems? Simulation studies suggest that hierarchical modular connectivity broadens the parameter space supporting critical-like behavior [48-50], but the exact mechanism, its universality, and its relation to the edge of chaos remain unclear.

Thus, we set out to understand how modular and hierarchical connectivity organization affects dynamics at different levels of the hierarchy. As we demonstrate below, the presence of modules gives rise to qualitatively distinct chaotic phases. High-dimensional chaotic activity (microscopic chaos) common in homogeneous networks [51,52] is separated from the low-dimensional chaotic activity of strongly coherent modules (macroscopic chaos) by an interesting crossover region (multiscale chaos), wherein both forms of chaotic activity coexist and the dimension of activity can be interpreted as either high or low, depending on the measure used. Furthermore, random hierarchical interaction structures significantly enhance the robustness of the edge of chaos without the need for precise fine tuning. The underlying mechanism is remarkably simple: Different levels of the hierarchy effectively compete for activity and the hierarchical organization in the network’s interactions coarse-grains chaotic fluctuations from lower levels to higher ones, preventing the amplification of chaos at higher levels in the hierarchy.

## Model—

We study a class of random network models commonly used in neuroscience [51], artificial intelligence [7,53], and ecology [54-56], incorporating modular and hierarchical organization into their interactions. We structure these interactions in levels, where higher levels are constructed by coarse-graining lower levels. Despite the generality of the model, for concreteness we refer to nodes of the network as neurons and to interaction strengths between them as synaptic weights. We start by introducing and analyzing a two-level modular system, in which the interaction matrix is generated in blocks corresponding to distinct populations, i.e., clusters of neurons with similar connectivity patterns. In effect, populations are introduced through a random and relatively low-rank connectivity perturbation [57,58] and, in the context of neuroscience, can be interpreted as emerging from shared spatial locations, morphologically or genetically defined cell types, or functions as in neuronal ensembles or engrams. Later we generalize our model and analysis by incorporating multiple levels.

Since we focus our attention on the effects of connectivity patterns, we choose a simple rate model with dynamics shared across all populations:

(1) x(t+1)=ϕ(Jx(t))

where x(t) is a vector of neural activity at time t, J is the connectivity matrix, and the activation function ϕ:R→R is applied elementwise. We take J to be a block matrix of the form

(2) $$J=\left[\begin{array}{cccc} J^{11} & J^{12} & \ldots & J^{1P}\\ J^{21} & J^{22} & \ldots & J^{2P}\\ \vdots & \vdots & \ddots & \vdots \\ J^{P1} & J^{P2} & \ldots & J^{\mathrm{PP}} \end{array}\right]$$

where each block $J^{\alpha \beta }$ is a submatrix that contains all synaptic weights from neurons in population β to all neurons in population α. For simplicity of notation, here we limit our attention to P populations of the same size n [59].

**FIG. 1.**
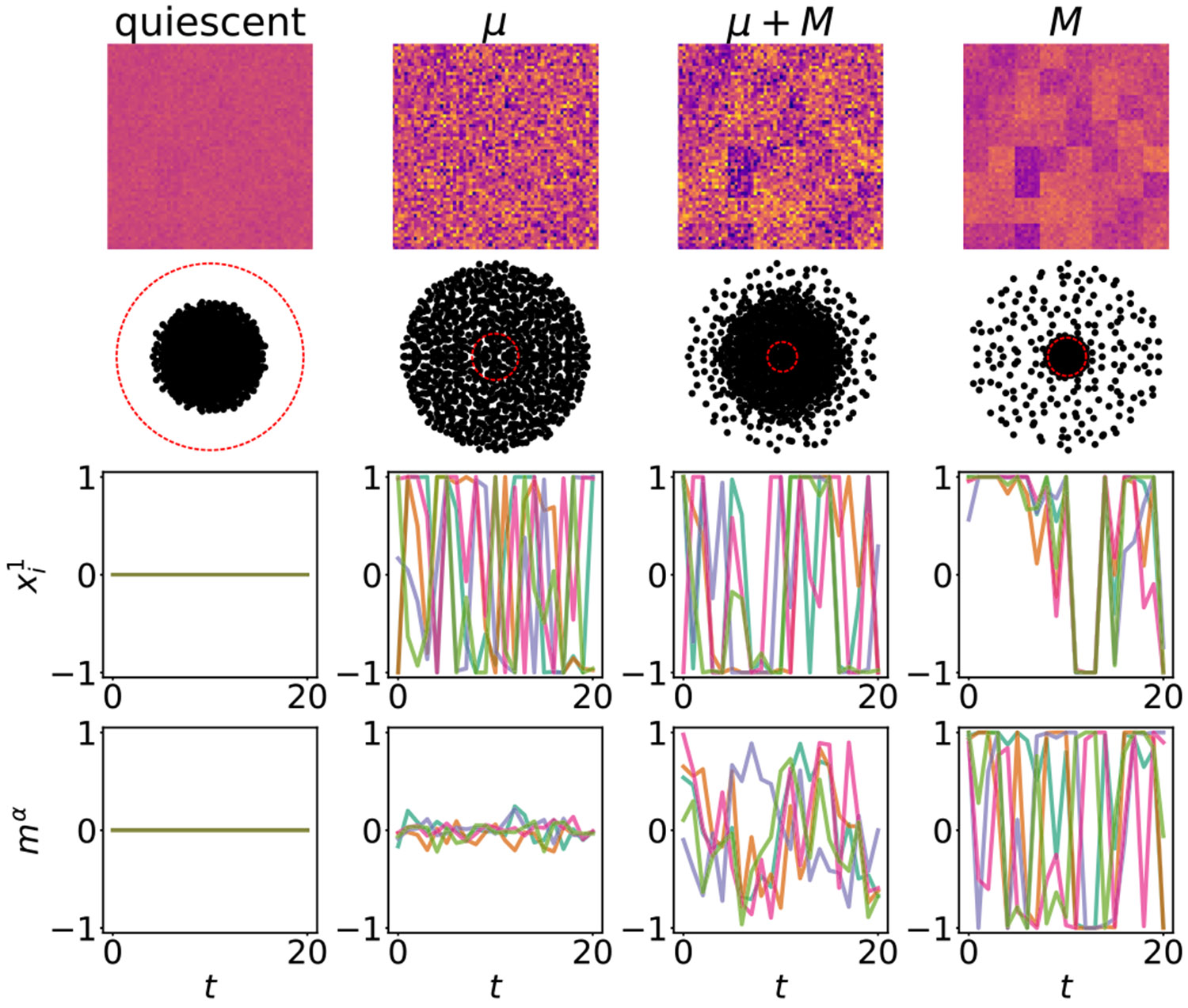
Visualizations of the weight matrix (top row), its eigenvalues (second row), and steady-state activities of five sample neurons from a shared population (third row) and five sample populations (bottom row) in four phases predicted by the mean-field theory: quiescent ($\sigma =\sigma_{\mu }=0.5$), μ (microscopic chaos, σ = 4, $\sigma_{\mu }$ = 0.5), μ+M (multiscale chaos, σ = 4, $\sigma_{\mu }$ = 6), and M (macroscopic chaos, σ = 1, $\sigma_{\mu }$ = 5). Red dashed lines in the eigenvalue spectra represent the unit circle in the complex plane.

To obtain a minimal and solvable model with characteristics of population-specific connectivity patterns, we define a hierarchical weight-generating process as follows. We endow each pair of populations (α, β) with a pair of parameters ($\mu^{\alpha \beta }$, $\sigma^{\alpha \beta }$) which are used to generate random weights between neurons in these populations as $J_{ij}^{\alpha \beta }∼𝒩(\mu^{\alpha \beta }∕n,\sigma^{\alpha \beta }∕\sqrt{n})$. These pairs of parameters are themselves generated randomly independent identically distributed (i.i.d.) from a specific distribution. In this work we focus our attention on the case of $\sigma^{\alpha \beta }=\sigma ∕\sqrt{P}$ fixed and shared across all pairs of populations and $\mu^{\alpha \beta }$ generated randomly as $\mu^{\alpha \beta }∼𝒩(0,\sigma_{\mu }∕\sqrt{P})$. The overall connectivity matrix can be concisely expressed as

(3) $$J=\sigma_{\mu }\Xi^{\left(P\right)}⊗O^{\left(n\right)}+\sigma \Xi^{\left(N\right)}$$

where ⊗ stands for the Kronecker product, N=nP is the total number of neurons, $O^{\left(n\right)}=vv^{T}$ is a fixed n×n orthogonal projection matrix with $v^{T}=(1∕\sqrt{n})[1\;1\;\ldots \;1]$, and $\Xi^{\left(m\right)}$ is a random m×m matrix with each entry generated i.i.d. from $𝒩(0,1∕\sqrt{m})$. Note that matrices $\Xi^{\left(P\right)}$ and $\Xi^{\left(N\right)}$ are mutually independent and correspond to different levels in the hierarchy of randomness: $\Xi^{\left(P\right)}$ to mean efficacies between pairs of populations and $\Xi^{\left(N\right)}$ to random fluctuations at the level of pairs of individual neurons. The resulting connectivity matrix has a block structure with a double-disk distribution of eigenvalues [58], see Fig. 1.

## Mean-field analysis—

In order to enable a straightforward mathematical analysis of the typical behavior of a network with multiple populations of neurons we consider a rather natural limit of a large number of large populations 1≪P≪N. The activity of neuron i in population α evolves according to Eq. (1), which we rewrite as

(4a) $$x_{i}^{\alpha }(t+1)=\phi (h_{i}^{\alpha }(t))$$

(4b) $$h_{i}^{\alpha }(t)=\sum_{\beta =1}^{P}\sum_{j=1}^{n}J_{ij}^{\alpha \beta }x_{j}^{\beta }(t)$$

where the input currents (preactivations) can be expressed as

(5) $$h_{i}^{\alpha }(t)=\sum_{\beta =1}^{P}\mu^{\alpha \beta }m^{\beta }(t)+\sum_{\beta =1}^{P}\sum_{j=1}^{n}\sigma \Xi_{ij}^{\alpha \beta }x_{j}^{\beta }(t)$$

with i.i.d. $\Xi_{ij}^{\alpha \beta }∼𝒩(0,1∕\sqrt{N})$ and where we define the mean activity of population α as

(6) $$m^{\alpha }(t)=\frac{1}{n}\sum_{i=1}^{n}x_{i}^{\alpha }(t).$$

When considered as a function of quenched synaptic weight disorder, the input current of each neuron is a Gaussian random variable. To keep track of the correlation structure between different neurons we rewrite it as a sum of population-specific and neuron-specific terms

(7) $$h_{i}^{\alpha }(t)=\sigma_{\mu }\sqrt{q_{m}(t)}{\tilde{z}}^{\alpha }+\sigma \sqrt{q(t)}z_{i}^{\alpha }$$

where ${\tilde{z}}^{\alpha }$, $z_{i}^{\alpha }$ are i.i.d. standard Gaussian random variables and

(8a) $$q_{m}(t)=\frac{1}{P}\sum_{\alpha =1}^{P}{\left[m^{\alpha }(t)\right]}^{2}$$

(8b) $$q(t)=\frac{1}{N}\sum_{\alpha =1}^{P}\sum_{i=1}^{n}{\left[x_{i}^{\alpha }(t)\right]}^{2}$$

are the order parameters that specify, respectively, the total activity variance, and the variance of mean population activities (macroscopic activity). In order to analyze the evolution of these order parameters we combine the single-neuron evolution equations (4) with Eqs. (7) and (8), average over quenched disorder, and obtain a coupled pair of mean-field equations (see Appendix A for the derivation)

(9a) $$q_{m}(t+1)=\int D\tilde{z}{\left[\int Dz\phi \left(\sigma_{\mu }\sqrt{q_{m}(t)}\tilde{z}+\sigma \sqrt{q(t)}z\right)\right]}^{2}$$

(9b) $$q(t+1)=\int Dz{\left[\phi \left(\sqrt{\sigma_{\mu }^{2}q_{m}(t)+\sigma^{2}q(t)}z\right)\right]}^{2}$$

where $\int Dzf(z)\equiv \int_{-\infty }^{\infty }dzf(z)\;\text{exp}(-z^{2}∕2)∕\sqrt{2\pi }$. Fixed points (q, $q_{m}$) of the mean-field equations (9) specify dynamical regimes (phases) of the network.

Before we proceed with the analysis of the mean-field equations, we note that the mean population activities behave like an effective recurrent neural network, with units corresponding to populations. Indeed, let us combine Eqs. (4)-(6), take the limit n→∞, and apply the law of large numbers, leading to

(10) $$m^{\alpha }(t+1)=\phi_{\sigma \sqrt{q}}\left(g(\sigma \sqrt{q})\sum_{\beta =1}^{P}\mu^{\alpha \beta }m^{\beta }(t)\right)$$

with $\phi_{a}(g(a)x)\equiv \int Dz\phi (x+az)$ and $g(a)\equiv \int Dz\phi^{\prime }(az)$ chosen such that $\phi_{a}^{\prime }(0)={[d\phi_{a}(x)∕dx]|}_{x=0}=1$. These considerations lead to a natural decomposition of network dynamics into two levels: macroscopic dynamics of means (activities of populations) and microscopic dynamics of neurons within populations around the means. A nonzero value of $q_{m}$ signals that the macroscopic dynamics is nonquiescent. We will refer to such dynamics as coherent, since in this case neural activities within populations are correlated. Similarly, a nonzero value of q signals that the overall activity of the network is nonquiescent. Finally, $q-q_{m}$ measures the average level of variability of neural activity within populations (microscopic dynamics).

In the following we assume that ϕ is continuous, odd, and nondecreasing with ϕ(∞)=1 and $\phi^{\prime }(0)=$. Thus, our results apply to the widely used tanh activation function, although in our computer simulations and some of the mathematical analysis we instead specialize to

(11) $$\phi_{\infty }(x)=\text{erf}\left(\frac{\sqrt{\pi }}{2}x\right).$$

This implies ϕ(0)=0, hence $q=q_{m}=0$ is a fixed point of the mean-field equations (9). The linearization around this fixed point

(12a) $$q_{m}(t+1)=\sigma_{\mu }^{2}q_{m}(t)$$

(12b) $$q(t+1)=\sigma_{\mu }^{2}q_{m}(t)+\sigma^{2}q(t)$$

demonstrates that the quiescent state with x=0 is stable for σ<1 and $\sigma_{\mu }<1$. Outside of this regime the fixed point with $q=q_{m}=0$ is unstable and a new stable fixed point with q>0 signals an active state, which can be subdivided into three qualitatively different phases and two associated phase transitions. The first transition is indicated by the bifurcation in the mean-field evolution equation of $q_{m}$ [Eq. (9a)] at $\sigma_{\mu }^{*}$ (for fixed σ). Below the transition $\left(\sigma_{\mu }<\sigma_{\mu }^{*}\right)$ the fixed point is such that $q_{m}=0$, translating into $m^{\alpha }(t)=0$ for all populations. In this phase activities within populations are not coherent. Above the transition $\left(\sigma_{\mu }>\sigma_{\mu }^{*}\right)$ the fixed point with $q_{m}=0$ is unstable and a stable fixed point with $q_{m}>0$ characterizes the steady state, translating into nonzero mean population activities (coherent chaos [26]). In order to derive an expression for $\sigma_{\mu }^{*}$, we linearize Eq. (9) around q>0 and $q_{m}=0$ and find

(13) $$\sigma_{\mu }^{*}=\frac{1}{g(\sigma \sqrt{q})}=\frac{1}{\int Dz\phi^{\prime }(\sigma \sqrt{q}z)}.$$

Although, as we show below, in this model the active state is necessarily chaotic, we can further distinguish two phases that differ in the nature and dimensionality of the chaotic activity manifold. When the value of $\sigma_{\mu }$ is relatively low, the corresponding chaotic attractor spans a high dimensional subspace and any random perturbation is rapidly expanded. In contrast, for $\sigma_{\mu }\gg \sigma$ the chaotic attractor is confined to a relatively low dimensional manifold and perturbations in random directions are contracted with high probability. The directions of expansion are aligned with the macroscopic activity.

Our derivation is based on the analysis of the evolution of an infinitesimal perturbation tracking the activity difference between two replicas with shared synaptic weights. As in Ref. [20], discrete-time dynamics and self-averaging allow us to infer the maximal Lyapunov exponent (MLE) from a single-step expansion rate of the perturbation, but here we need to decompose the perturbation into two components: $\epsilon_{i}^{\alpha }(t)=r_{i}^{\alpha }(t)+\delta m^{\alpha }(t)$, with macroscopic perturbation $\delta m^{\alpha }(t)$ lying in a P-dimensional subspace of the N-dimensional neural activity space and microscopic perturbation $r_{i}^{\alpha }(t)$ lying in the (N−P)-dimensional orthogonal complement defined by P constraints $\sum_{i}r_{i}^{\alpha }(t)=0$. The linearization of Eq. (4) around ϵ(t)=0 gives

(14) $$\epsilon_{i}^{\alpha }(t+1)=\phi^{\prime }\left(\sum_{j,\beta }J_{ij}^{\alpha \beta }x_{j}^{\beta }(t)\right)\sum_{j^{\prime },\beta^{\prime }}J_{ij^{\prime }}^{\alpha \beta^{\prime }}\epsilon_{j^{\prime }}^{\beta^{\prime }}(t)$$

where, as before, $J_{ij}^{\alpha \beta }=(\sigma_{\mu }∕\sqrt{P})(1∕n){\tilde{z}}^{\alpha \beta }+(\sigma ∕\sqrt{N})z_{ij}^{\alpha \beta }$. To summarize the evolution of the perturbation within each subspace we define $q^{\epsilon }(t)=(1∕N){\left\|\epsilon (t)\right\|}^{2}$ and $q_{m}^{\epsilon }(t)=(1∕N){\left\|\delta m(t)\right\|}^{2}$. In words, $q_{m}^{\epsilon }(t)$ and $q^{\epsilon }(t)-q_{m}^{\epsilon }(t)$ denote normalized squared lengths of the perturbation at time t within, respectively, macroscopic and microscopic subspaces. We rewrite Eq. (14) as $\epsilon_{i}^{\alpha }(t+1)=\eta_{i}^{\alpha }(t)\phi^{\prime }(h_{i}^{\alpha }(t))$ where Gaussian fields $h_{i}^{\alpha }(t)=\sigma_{\mu }\sqrt{q_{m}}{\tilde{z}}^{\alpha }+\sigma \sqrt{q}z_{i}^{\alpha }$ and $\eta_{i}^{\alpha }(t)=\sigma_{\mu }\sqrt{q_{m}^{\epsilon }(t)}{\tilde{\zeta }}^{\alpha }+\sigma \sqrt{q^{\epsilon }(t)}\zeta_{i}^{\alpha }$ are assumed to be independent (i.e., $z_{i}^{\alpha }$, ${\tilde{z}}^{\alpha }$, $\zeta_{i}^{\alpha }$, ${\tilde{\zeta }}^{\alpha }$ are independent standard Gaussian random variables), which follows from the observation that x(t) and ϵ(t) are generically expected to be orthogonal. Finally, we average over the Gaussian fields, leading to (see Appendix B for the derivation)

(15) $$\left(\begin{array}{c} q_{m}^{\epsilon }(t+1)\\ q^{\epsilon }(t+1)-q_{m}^{\epsilon }(t+1) \end{array}\right)=D\left(\begin{array}{c} q_{m}^{\epsilon }(t)\\ q^{\epsilon }(t)-q_{m}^{\epsilon }(t) \end{array}\right)$$

with

(16) $$D=\left(\begin{array}{cc} R_{\text{coherent}}^{2} & 0\\ C & R_{\text{random}}^{2} \end{array}\right)$$

where $C=(\sigma_{\mu }^{2}∕\sigma^{2}+1)R_{\text{random}}^{2}-R_{\text{coherent}}^{2}\geq 0$ and

(17a) $$R_{\text{coherent}}^{2}=\sigma_{\mu }^{2}\int D\tilde{z}{\left[\int Dz\phi^{\prime }(\sigma_{\mu }\sqrt{q_{m}}\tilde{z}+\sigma \sqrt{q}z)\right]}^{2}$$

(17b) $$R_{\text{random}}^{2}=\sigma^{2}\int Dz{\left[\phi^{\prime }\left(\sqrt{\sigma_{\mu }^{2}q_{m}+\sigma^{2}q}z\right)\right]}^{2}$$

are the eigenvalues of the D. The long-term evolution of a generic perturbation will be dominated by the larger of the two. Therefore, the MLE can be calculated as $\lambda_{\max}=\max(\lambda_{\text{random}},\lambda_{\text{coherent}})$ where $\lambda_{X}=\text{ln}(R_{X})$.

The interactions between macroscopic and microscopic dynamics are asymmetric. Although a macroscopic perturbation has a direct effect on microscopic activity, a microscopic perturbation does not transmit to macroscopic activity due to averaging. This translates into the lower-triangular form of D. Moreover, high-dimensional chaos resulting from chaotic microscopic dynamics can be suppressed by high levels of macroscopic activity. Indeed, the asymptotic behavior of $R_{\text{random}}^{2}$ for $\sigma_{\mu }\gg 1$ reads $R_{\text{random}}^{2}\approx C_{0}\sigma^{2}∕\sqrt{\sigma_{\mu }^{2}q_{m}+\sigma^{2}q}$. Since $q_{m}$ and q are nondecreasing and bounded functions of $\sigma_{\mu }$, for any fixed σ there exists $\sigma_{\mu }^{\text{EoHC}}$ such that for $\sigma_{\mu }>\sigma_{\mu }^{\text{EoHC}}$ a random perturbation within the (N−P)-dimensional microscopic subspace is contracted $\left(R_{\text{random}}^{2}<1\right)$, i.e., high-dimensional chaos is suppressed. Based on this analysis, we can expect that around $\sigma_{\mu }^{\text{EoHC}}$ the system will transition between low-dimensional and high-dimensional chaos.

## Computer simulations—

**FIG. 2.**
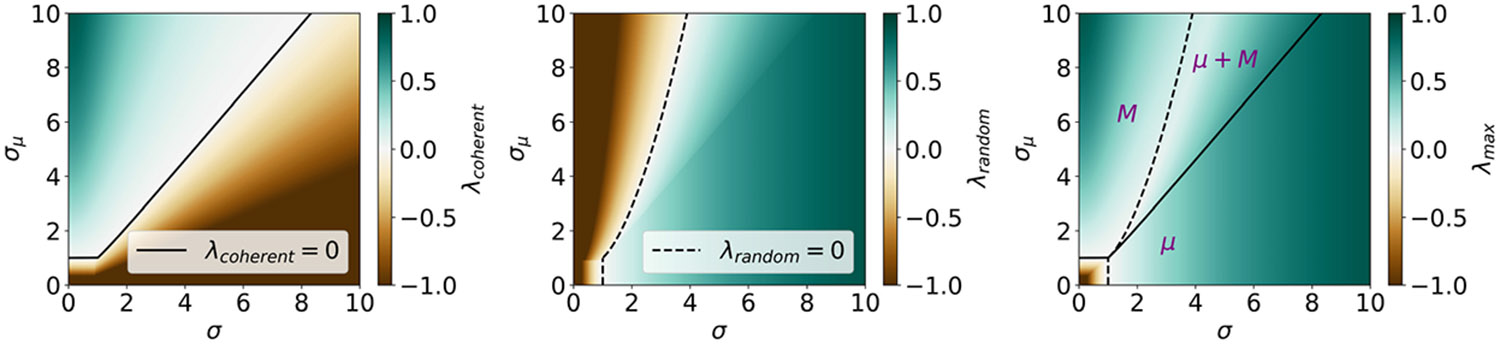
The mean-field phase diagram. Lines represent transition lines: $\sigma_{\mu }^{*}$ (solid) and $\sigma_{\mu }^{\mathrm{EoHC}}$ (dashed). Phases are denoted by symbols M (macroscopic chaos) and μ (microscopic chaos). Heat maps represent the values of $\lambda_{\text{coherent}}$ (left), $\lambda_{\text{random}}$ (center), and $\lambda_{\text{max}}$ (right).

**FIG. 3.**
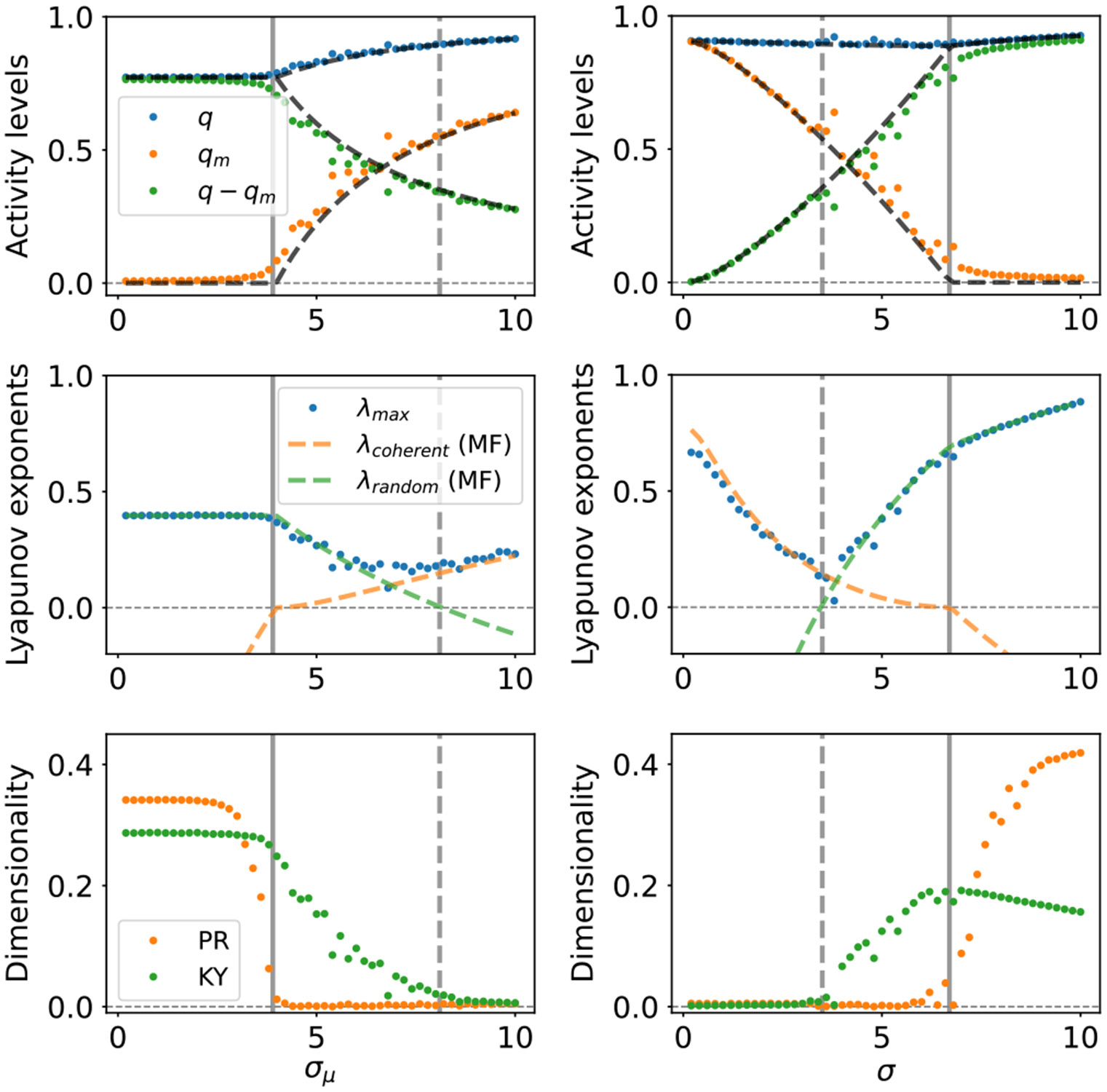
Statistics of the typical autonomous dynamics as functions of σ for $\sigma_{\mu }$ = 3.5 (left) and as functions of σ for $\sigma_{\mu }$ = 8 (right). Dots and dashed lines correspond to results of computer simulations and predictions of the mean-field theory, respectively. Vertical gray lines represent transition lines: $\lambda_{\text{coherent}}=0$ (solid) and $\lambda_{\text{random}}=0$ (dashed). Top: Total (q), macroscopic ($q_{m}$), and microscopic ($q-q_{m}$) variances. Center: Maximal Lyapunov exponents computed either directly from simulations or as within-subspace theoretical predictions. Bottom: normalized dimensionality of the steady-state neural activity manifold as measured by the covariance matrix (PR dimension) or Lyapunov exponents (KY dimension).

We test this prediction with computer simulations (see Appendix E for details). In Fig. 1, we present eigenvalues of the weight matrix and example activities of neurons and populations in the four phases predicted by the mean-field theory. In Fig. 3, we illustrate relevant statistics of the dynamics as functions of control parameters. As expected, with fixed σ>1 and increasing $\sigma_{\mu }$, we observe a region with a substantial drop in the values of the Kaplan-Yorke (KY or Lyapunov) dimension [60-62], where the Lyapunov dimension transitions from being extensive in N to being extensive in P. In contrast, the participation ratio (PR) dimension [52,63-66] exhibits an abrupt transition around $\sigma_{\mu }^{*}$, leading to a region where these two measures of attractor dimensionality differ by orders of magnitude. A similar region is observed with fixed $\sigma_{\mu }>1$ and increasing σ. Additionally, in this case the Lyapunov dimension features a peak and slowly decreases at large values of σ. This effect has been previously noted and explained in the single-population model [67]. Other quantitative predictions of our mean-field analysis are also confirmed by the simulations. In particular, the MLE admits a minimum at intermediate values of σ (for fixed $\sigma_{\mu }$) or $\sigma_{\mu }$ (for fixed σ), suggestive of an effective competition between macroscopic and microscopic dynamics. In other words, both adding modular structure (increasing $\sigma_{\mu }$) to mostly random connectivity (large initial σ and small initial $\sigma_{\mu }$) and injecting noise (increasing σ) to strongly modular connectivity (large initial $\sigma_{\mu }$ and small σ) lead to attenuation of chaos. These results are summarized as a phase diagram in Fig. 2. Furthermore, our preliminary simulations confirm that the same qualitative results are obtained in the continuous-time version of the model [68].

## Multilevel generalization—

In biological neural circuits we expect each population to consist of multiple subpopulations, which in turn may be further subdivided into sub-subpopulations, and so on. To capture such a hierarchical connectivity organization we introduce a generalization of our model in the following, recursive manner:

(18) $$J^{\left[i\right]}=J^{\left[i-1\right]}⊗O^{\left(P_{i}\right)}+\sigma_{i}\Xi^{\left(N_{i}\right)}$$

where $N_{i}=\prod_{j=0}^{i}P_{j}$, $P_{0}=1,J^{\left[0\right]}=0$, and $\sigma_{i}$ and $P_{i}$ are parameters specific to level i. Our basic model is equivalent to the case with two levels $\left(J=J^{\left[2\right]}\right)$ and $\sigma_{1}=\sigma_{\mu }$, $\sigma_{2}=\sigma$, $P_{1}=P$, $P_{2}=n$.

To facilitate the analysis we make use of tensor notation with indices that specify the unit at each level of the hierarchy. We rewrite the evolution equation as $x_{\alpha_{1}\ldots \alpha_{L}}(t+1)=\phi (h_{\alpha_{1}\ldots \alpha_{l}}(t))$ with $h_{\alpha_{1}\ldots \alpha_{L}}(t)=\sum_{\beta_{1},\ldots ,\beta_{L}}J_{\alpha_{1}\ldots \alpha_{L}}^{\beta_{1}\ldots \beta_{L}}x_{\beta_{1}\ldots \beta_{L}}(t)$, and introduce mean activities at level j as $m_{\alpha_{1}\ldots \alpha_{j}}(t)=(N_{j}∕N_{L})\sum_{\alpha_{j+1},\ldots ,\alpha_{L}}x_{\alpha_{1}\ldots \alpha_{L}}(t)$. Since we only make use of sums over adjacent right-most indices, this simplified notation does not introduce any ambiguity. We assume $\forall_{i}P_{i}\gg 1$ and substitute $\sum_{j=1}^{L}\sigma_{j}\sqrt{q_{j}(t)}z_{\alpha_{1}\alpha_{2}\ldots \alpha_{j}}^{\left[j\right]}$ for the preactivations $h_{\alpha_{1}\ldots \alpha_{L}}(t)$, where $z_{\alpha_{1}\ldots \alpha_{j}}^{\left[j\right]}$ are i.i.d. standard normal random variables and $q_{j}(t)=(1∕N_{j})\sum_{\alpha_{1},\ldots ,\alpha_{j}}{\left(m_{\alpha_{1}\ldots \alpha_{j}}(t)\right)}^{2}$ are the order parameters that in effect evolve as (see Appendix A for the derivation)

(19) $$q_{j}(t+1)=\int D\tilde{z}{\left[\int Dz\phi \left(\sqrt{A_{j}(t)}\tilde{z}+\sqrt{A_{L}(t)-A_{j}(t)}z\right)\right]}^{2}$$

where $A_{j}(t)=\sum_{i=1}^{j}\sigma_{i}^{2}q_{i}(t)$. As in the two-level case, here also, each level contributes its own (maximal within the relevant subspace) Lyapunov exponent $\lambda_{j}=\ln\;R_{i}$, which in the mean-field limit can be expressed as (see Appendix B for the derivation)

(20) $$R_{j}^{2}=\sigma_{j}^{2}\int D\tilde{z}{\left[\int Dz\phi^{\prime }\left(\sqrt{A_{j}}\tilde{z}+\sqrt{A_{L}-A_{j}}z\right)\right]}^{2}$$

where $A_{j}=\lim_{t\to \infty }\;A_{j}(t)$. In the special case of $\phi (x)=\phi_{\infty }(x)$ we evaluate all relevant integrals and arrive at closed-form expressions

(21) $$\sigma_{j}^{2}=\frac{2}{\pi q_{j}}\frac{\sin\;(\frac{\pi }{2}\;q_{j})-\sin\;(\frac{\pi }{2}\;q_{j-1})}{1-\sin\;(\frac{\pi }{2}\;q_{L})}$$

and

(22) $$R_{j}^{2}=\frac{2}{\pi q_{j}}\frac{\sin\;(\frac{\pi }{2}\;q_{j})-\sin\;(\frac{\pi }{2}\;q_{j-1})}{\cos\;(\frac{\pi }{2}\;q_{j})}.$$

Equation (21) specifies a sequence of control parameters that gives rise to a steady state described by an admissible sequence of order parameters $0=q_{0}\leq q_{1}\leq q_{2}\leq \ldots \leq q_{L}<1$ [69].

Given the explicit form of Eq. (22), we are ready to investigate how the MLE depends on the levels of network activity. Let k be such that $q_{k-1}=0$ and $q_{k}>0$. The corresponding MLE $\lambda_{k}=\frac{1}{2}\ln\{\tan[(\pi ∕2)q_{k}]∕[(\pi ∕2)q_{k}]\}$ is positive and does not depend on the order parameters at lower levels. Thus, the nonquiescent steady state of this model is always chaotic. However, in deep hierarchical structures with L≫1 we can expect the overall MLE to be close to zero, as long as many levels are active and approximately balanced, i.e., if $\Delta_{j}=q_{j}-q_{j-1}$ are all of the order of $q_{L}∕L$. For L≫1 we then have $\Delta_{j}\ll 1$ and we can expand the level-specific MLEs as $\lambda_{k}=\pi^{2}\Delta_{k}^{2}∕24$ and, for j>k, $\lambda_{j}\approx \frac{1}{2}\ln((\Delta_{j}∕q_{j})+\{\pi \;\tan[(\pi ∕2)q_{j}]∕4q_{j}\}\Delta_{j}^{2})$ [70]. This demonstrates that $\lambda_{\max}\approx 0\;\text{for}\;L\gg 1\;\text{and}\;L^{2}\overset{>}{\approx }\tan[(\pi ∕2)q_{L}]$. Therefore, any mechanism that maintains a loosely balanced hierarchical activity of the network (i.e., with all $\Delta_{j}$ of the order of 1∕L) will also keep it close to the edge of chaos. To illustrate this, we devise and test a proof-of-concept adaption algorithm [71]. Importantly, although our analysis above assumes $\phi (x)=\phi_{\infty }(x)$, we expect its conclusions to be more general since, due to an effective coarse-graining, the dynamics at higher levels of the hierarchy is universal for a wide range of activation functions (see Appendix D for details).

## Conclusion—

We introduced a modular neural network model and studied its autonomous dynamics. We showed that, with increasing correlations between within-population weights, the suppression of high-dimensional chaotic dynamics proceeds in two steps. First, starting from $\sigma_{\mu }^{*}$ the macroscopic activity of populations becomes coherent, as signaled by nonzero $q_{m}$, positive $\lambda_{\text{coherent}}$, and a low value of the participation ratio dimension. At $\sigma_{\mu }^{*}$, critical macroscopic dynamics coexist with noncritical, chaotic microscopic dynamics, analogous to the low-dimensional criticality embedded in high-dimensional neural dynamics recently uncovered in the motor cortex of awake, behaving mice [72]. Second, high-dimensional chaos is completely suppressed above $\sigma_{\mu }^{\mathrm{EoHC}}$, as signaled by negative $\lambda_{\text{random}}$ and a low value of the Lyapunov dimension. Moreover, the maximal Lyapunov exponent features a prominent dip at intermediate values of weight correlations, indicating that modular synaptic connectivity supports dynamics in the vicinity of the edge of chaos and suggesting an interesting computational regime that maintains the balance between macroscopic and microscopic dynamics. This effect is even more pronounced in the multilevel generalization of the model. We hypothesize that it provides an example of a general mechanism that underpins a potentially ubiquitous phenomenon of complex biological systems evolving towards the edge of chaos through a loose balance of multiscale, hierarchical dynamics. Furthermore, brain activity at different levels of hierarchy may encode and process distinct information, with level-specific coherent-quiescent transitions providing a substrate for the flexible modulation of information flow.

## Supplementary Material

Supplementary Material

## Data Availability

The data that support the findings of this Letter are openly available [73].

## Appendix A: Derivation of Eq. (19)—

As noted in the main text, we rewrite the evolution equation as

(A1)
$$x_{\alpha_{1}\ldots \alpha_{L}}(t+1)=\phi \left(\sum_{j=1}^{L}\sigma_{j}\sqrt{q_{j}(t)}z_{\alpha_{1}\ldots \alpha_{j}}^{\left[j\right]}\right)$$

where $z_{\alpha_{1}\alpha_{2}\ldots \alpha_{j}}^{\left[j\right]}∼𝒩(0,1)$ are i.i.d. This decomposition originates from the hierarchical structure of the weight matrix, which contains a sum over L levels, each contributing an independent source of variability:

(A2)
$$J_{\alpha_{1}\ldots \alpha_{L}}^{\beta_{1}\ldots \beta_{L}}=\sum_{j=1}^{L}\frac{1}{P_{j+1}\ldots P_{L}}\frac{\sigma_{j}}{\sqrt{N_{j}}}{\tilde{z}}_{\alpha_{1}\ldots \alpha_{j}\beta_{1}\ldots \beta_{j}}^{\left[j\right]}$$

where ${\tilde{z}}_{\alpha_{1}\ldots \alpha_{j}\beta_{1}\ldots \beta_{j}}^{\left[j\right]}∼𝒩(0,1)$ are all independent. Equation (A1) follows immediately from

(A3)
$$\sum_{\beta_{1}\ldots \beta_{L}}\frac{1}{P_{j+1}\ldots P_{L}}\frac{1}{\sqrt{N_{j}}}{\tilde{z}}_{\alpha_{1}\ldots \alpha_{j}\beta_{1}\ldots \beta_{j}}^{\left[j\right]}x_{\beta_{1}\ldots \beta_{L}}(t)=\sqrt{q_{j}(t)}z_{\alpha_{1}\ldots \alpha_{j}}^{\left[j\right]}.$$

Our goal is to find an evolution equation of

(A4)
$$q_{j}(t)=\frac{1}{P_{1}\ldots P_{j}}\sum_{\alpha_{1},\ldots ,\alpha_{j}}{\left(m_{\alpha_{1}\ldots \alpha_{j}}(t)\right)}^{2}$$

where

(A5)
$$m_{\alpha_{1}\ldots \alpha_{j}}(t)=\frac{1}{P_{j+1}\ldots P_{L}}\sum_{\alpha_{j+1},\ldots ,\alpha_{L}}x_{\alpha_{1}\ldots \alpha_{L}}(t).$$

We start by rewritting Eq. (A5) at time t+1 using Eq. (A1)

(A6)
$$m_{\alpha_{1}\ldots \alpha_{j}}(t+1)=\frac{1}{P_{j+1}\ldots P_{L}}\sum_{\alpha_{j+1},\ldots ,\alpha_{L}}\phi \left(\sum_{i=1}^{L}\sigma_{i}\sqrt{q_{i}(t)}z_{\alpha_{1}\ldots \alpha_{i}}^{\left[i\right]}\right).$$

We note that all terms under the activation function with i>j contain at least a single index $\alpha_{k}$ that is averaged over, which in the limit of $P_{k}\to \infty$ converges to the expectation over the corresponding standard normal variable. In contrast, terms with i≤j do not contain summation indices $\alpha_{j+1},\ldots ,\alpha_{L}$ and are not affected by the averaging. Thus, the right-hand side (rhs) of Eq. (A6) can be rewritten as

(A7)
∫RL−jDzj+1…DzLϕ(∑i=1jσiqi(t)zα1…αi[i])+(∑k=j+1Lσkqk(t)zk)

where $Dz_{i}=dz_{i}\;\exp(-z_{i}^{2}∕2)∕\sqrt{2\pi }$. Due to the stability of the Gaussian distribution (convolutions of Gaussian distributions are still Gaussian), we can replace the expectation of a function of a sum of Gaussian random variables with an expectation of the same function over a properly scaled single Gaussian random variable, i.e.,

(A8)
$$\int_{R^{n}}Dz_{1}\ldots Dz_{n}f\left(\sum_{i=1}^{n}a_{i}z_{i}\right)=\int_{R}Dzf\left(z\sqrt{\sum_{i=1}^{n}a_{i}^{2}}\right),$$

reducing Eq. (A6) to

(A9)
mα1…αj(t+1)=∫RDzϕ(∑i=1jσiqi(t)zα1…αi[i])+(AL(t)−Aj(t)z)

where $A_{k}(t)=\sum_{i=1}^{k}\sigma_{i}^{2}q_{i}(t)$. In the final step we plug Eq. (A9) into Eq. (A4), repeat the process of replacing averages with expected values, and subsequently replacing expectations over multiple Gaussian random variables with an expected value over a single Gaussian random variable.

## Appendix B: Derivation of Eq. (20)—

Similarly to the basic two-level model, we introduce an infinitesimal perturbation $\epsilon_{\alpha_{1}\ldots \alpha_{L}}(t)$ and linearize the evolution equation (4), arriving at a linear evolution equation for the perturbation

(B1)
$$\epsilon_{\alpha_{1}\ldots \alpha_{L}}(t+1)=\phi^{\prime }\left(\sum_{\beta_{1},\ldots ,\beta_{L}}J_{\alpha_{1}\ldots \alpha_{L}}^{\beta_{1}\ldots \beta_{L}}x_{\beta_{1}\ldots \beta_{L}}(t)\right)\;\;\times \sum_{\gamma_{1},\ldots ,\gamma_{L}}J_{\alpha_{1}\ldots \alpha_{L}}^{\gamma_{1}\ldots \gamma_{L}}\epsilon_{\gamma_{1}\ldots \gamma_{L}}(t).$$

In analogy to the order parameters used to network activity, we introduce

(B2)
$$m_{\alpha_{1}\ldots \alpha_{j}}^{\epsilon }(t)=\frac{1}{P_{j+1}\ldots P_{L}}\sum_{\alpha_{j+1},\ldots ,\alpha_{L}}\epsilon_{\alpha_{1}\ldots \alpha_{L}}(t)$$

(B3)
$$q_{j}^{\epsilon }(t)=\frac{1}{P_{1}\ldots P_{j}}\sum_{\alpha_{1},\ldots ,\alpha_{j}}{\left(m_{\alpha_{1}\ldots \alpha_{j}}^{\epsilon }(t)\right)}^{2}.$$

We replace both sums in Eq. (B1) with Gaussian fields

(B4)
$$\epsilon_{\alpha_{1}\ldots \alpha_{L}}(t+1)=\phi^{\prime }\left(\sum_{j=1}^{L}\sigma_{j}\sqrt{q_{j}(t)}z_{\alpha_{1}\ldots \alpha_{j}}^{\left[j\right]}\right)\;\;\times \sum_{k=1}^{L}\sigma_{k}\sqrt{q_{k}^{\epsilon }(t)}{\tilde{z}}_{\alpha_{1}\ldots \alpha_{k}}^{\left[k\right]}.$$

Crucially, we assume that $z_{\alpha_{1}\ldots \alpha_{j}}^{\left[j\right]}$ and ${\tilde{z}}_{\alpha_{1}\ldots \alpha_{j}}^{\left[j\right]}$ are independent, which follows from the assumption that x(t) and ϵ(t) are generically orthogonal within any of the considered subspaces (at each level of coarse-graining). Given this assumption, we can replace averages with expectations over independent Gaussian random variables. After straightforward manipulations we arrive at

(B5)
qjϵ(t+1)=Ajϵ(t)∫−∞∞Dz~(∫−∞∞Dzϕ′(Aj(t)z~))+((AL(t)+Aj(t)z))2

where $A_{j}^{\epsilon }(t)=\sum_{i=1}^{j}\sigma_{i}^{2}q_{i}^{\epsilon }(t)$ comes from the term $\sum_{k=1}^{L}\sigma_{k}\sqrt{q_{k}^{\epsilon }(t)}{\tilde{z}}_{\alpha_{1}\ldots \alpha_{k}}^{\left[k\right]}$. In the steady state, $q_{j}=\lim_{t\to \infty }\;q_{j}(t)$ and $A_{j}=\lim_{t\to \infty }\;A_{j}(t)$ are constant and Eq. (B5) is linear dynamical system of the form $q^{\epsilon }(t+1)={\mathrm{Dq}}^{\epsilon }(t)$, where D is an L×L time-independent triangular matrix. Its diagonal entries correspond exactly to its eigenvalues and are given by Eq. (20).

## Appendix C: Relevant integrals—

In order to derive Eqs. (21) and (22) from Eqs. (19) and (20) we used the following identities:

(C1)
$$\int_{R}Dz\phi_{\infty }(az+b)=\phi_{\infty }\left(\frac{b}{\sqrt{1+\pi a^{2}∕2}}\right)$$

(C2)
$$\int_{R}Dz{\left[\phi_{\infty }(cz)\right]}^{2}=\frac{4}{\pi }\arctan\left(\sqrt{1+\pi c^{2}}\right)-1.$$

To prove Eq. (C1), we introduce $f(a,b)=\int_{R}Dz\phi_{\infty }(az+b)$ and calculate $\partial_{b}f(a,b)$. Since $\phi_{\infty }^{\prime }=\exp(-\pi x^{2}∕4)$, this results in a Gaussian integral (over the entire real line) which is straightforward to evaluate, leading to

(C3)
$$\partial_{b}f(a,b)=\frac{1}{\sqrt{1+\frac{\pi }{2}a^{2}}}\;\text{exp}\left(-\frac{\frac{\pi }{4}b^{2}}{1+\frac{\pi }{2}a^{2}}\right).$$

Thus, f(a,b) is given by the indefinite Gaussian integral

(C4)
$$f(a,b)=\phi_{\infty }\left(\frac{b}{\sqrt{1+\pi a^{2}∕2}}\right)+C(a)$$

where C(a) is an unknown function of a. Finally, due to the fact that $\phi_{\infty }$ is odd we find that C(a)=f(a,0)=0.

To prove Eq. (C2), we introduce $g(c)=\int Dz{\left[\phi_{\infty }(cz)\right]}^{2}$ and calculate its derivative

(C5)
$$g^{\prime }(c)=\sqrt{\frac{2}{\pi }}\int_{-\infty }^{\infty }dz\;\text{exp}\left[-\frac{z^{2}}{2}\left(1+\frac{\pi }{2}c^{2}\right)\right]z\phi_{\infty }(cz).$$

We rewrite the rhs of Eq. (C5) as

(C6)
$$\sqrt{\frac{2}{\pi }}\int_{-\infty }^{\infty }dz\frac{d}{dz}\left\{-\frac{1}{1+\frac{\pi }{2}c^{2}}\;\text{exp}\left[-\frac{z^{2}}{2}\left(1+\frac{\pi }{2}c^{2}\right)\right]\right\}\phi_{\infty }(cz)$$

and, applying integration by parts, arrive at

(C7)
$$g^{\prime }(c)=\frac{2c}{\left(1+\frac{\pi }{2}c^{2}\right)\sqrt{1+\pi c^{2}}}.$$

Next, we set $u=\sqrt{1+\pi c^{2}}$ and perform integration by substitution, which gives

(C8)
$$g(c)=\frac{4}{\pi }\arctan(\sqrt{1+\pi c^{2}})+C_{0}.$$

To find the value of $C_{0}$, we observe that g(0)=0 [since $\phi_{\infty }(0)=0$], from which we infer $C_{0}=-1$.

## Appendix D: Universality—

Assume ϕ to be odd with ϕ(∞)=1 and a single-peaked $\phi^{\prime }$. If most lower levels contribute to the activity of the multilevel system, we have $\sigma_{i}>1$ for most levels. Thus, at high-enough levels of the hierarchy, j≪L, we have $A_{L}-A_{j}\gg 1$ and the limit $\lim_{a\to \infty }a\phi^{\prime }(ax)=2\delta (x)$ provides a way of approximating Eqs. (19) and (20) as

(D1)
$$q_{j}(t+1)\approx \int Dz{\left[\phi_{\infty }(B_{j}(t)z)\right]}^{2}$$

and

(D2)
$$R_{j}^{2}\approx {\tilde{\sigma }}_{j}^{2}\int Dz{\left[\phi_{\infty }^{\prime }(B_{j}z)\right]}^{2},$$

where ${\tilde{\sigma }}_{j}^{2}=(2∕\pi )[\sigma_{j}^{2}∕(A_{L}-A_{j})]$, $B_{j}(t)=\sqrt{\left(2∕\pi \right)}\sqrt{\left\{A_{j}(t)∕[A_{L}(t)-A_{j}(t)]\right\}}$, $B_{j}=\lim_{t\to \infty }\;B_{j}(t)$, and $\phi_{\infty }(x)=\lim_{a\to \infty }\;\phi_{a}(x)$ is given by (11). Because of Eq. (C1), the two formulas above match the mean-field equations for a multilevel system with the activation function set to $\phi_{\infty }$. Indeed, direct computations similar to Eq. (10) confirm that $\phi_{\infty }$ is the activation function of the effective evolution of the higher level activities. In other words, $\phi_{\infty }$ is a fixed point of the coarse-graining procedure for a wide range of underlying microscopic activation functions. This illustrates the special status of $\phi_{\infty }$ as the effective activation function of higher, population level activities, and it provides a justification for the expectation that our results extend with no qualitative changes to many activation functions.

## Appendix E: Details of the computer simulations—

Figure 1: For visualization purposes, network sizes differed between weight visualizations (*n* = 8, *P* = 8), eigenvalues panels (*n* = 5, *P* = 200), and the simulations (*n* = 200, *P* = 100). Networks were simulated for 220 steps starting from random initial conditions, with activities from the last 20 steps used for plotting. The random seed was shared across columns. In Fig. 3, recurrent neural networks with *P* = *n* = 100 were simulated for 22 000 steps starting from random initial conditions [i.i.d. $x_{i}^{\alpha }∼𝒩(0,1)$]. Weights and initial conditions were reinitialized for each pair of values of control parameters (σ, $\sigma_{\mu }$). In order to ensure that the statistics were collected close to the steady state, the first 2000 steps were discarded. Network activity at all the remaining 20 000 steps was used to calculate q, $q_{m}$, and the participation ratio dimension. Network activity during the last 500 steps was used to compute Lyapunov exponents and the associated Lyapunov dimension. Dimensionalities were normalized by the total number of neurons (10^4^). For numerical stability, QR decomposition was used in computations of the Lyapunov exponents; for details of the algorithm see Ref. [74]. The code, available online [73], was written in python [75] utilizing jax [76] for GPU acceleration.
