# Supplementary material for: Hierarchy of Chaotic Dynamics in Random Modular Networks
Source: Phys Rev Lett. Author manuscript; Available in PMC 2026 May 21. (PMC13193674; doi:10.1103/PhysRevLett.134.148402)
Supplement: Supplementary Material [file NIHMS2171662-supplement-Supplementary_Material.pdf]

Supplementary Material: Hierarchy of chaotic dynamics in random modular networks

Łukasz Kuśmierz,[1] Ulises Pereira-Obilinovic,[1] Zhixin Lu,[1] Dana Mastrovito,[1] and Stefan Mihalas[1]
[1]Allen Institute, Seattle, WA

I. $\sigma(\Delta)$ ADAPTATION ALGORITHM

In the main text, we argue that an adaptation algorithm driving the system toward a balance across hierarchical activity levels naturally positions it near the edge of chaos. To support this claim, here we introduce and test a simple adaptation algorithm. It is important to note that the algorithm presented here serves as a proof of concept rather than a specific model that we propose the brain, or any natural system, implements.

The algorithm is based on the observation that a hierarchical modular system with L levels should be close to the edge of chaos as long as $\Delta_i = q_i - q_{i-1}$ are all of the order of q_L/L, where q_L is the total activity variance of the network. Thus, given a desired total level of activity $\hat{q}_L$, we set the desired within-level activities to

$$\hat{\Delta}_i = \frac{\hat{q}_L}{L} \tag{1}$$

We assume that the synaptic weights are drawn randomly according to the multilevel version of our connectivity model (18), but with a time-dependent level-specific control parameters $\sigma_i(t)$, i.e.

$$\boldsymbol{J}^{[i]}(t) = \boldsymbol{J}^{[i-1]}(t) \otimes \boldsymbol{O}^{(P_i)} + \sigma_i(t)\Xi^{(N_i)} \tag{2}$$

For simplicity, the individual entries $\Xi^{(N_i)}$ are fixed. At each time step of the adaptation process, $\Delta_i(t)$ are computed based on the network activity $\boldsymbol{x}(t)$, and $\sigma_i(t)$ are updated according to

$$\sigma_i(t+1) = \sigma_i(t) + \eta\left(\hat{\Delta}_i - \Delta_i(t)\right) \tag{3}$$

where η is a fixed learning rate. Equation (3) was not derived from any cost function. Instead, it is based on the intuitive observation that, all else being equal, Δ_i is expected to be a non-decreasing function of σ_i. Note also that our choice of $\hat{\Delta}_i$ is somewhat arbitrary and is not actually optimal, i.e. it does not minimize the maximal Lyapunov exponent for a given $\hat{q}_L$. Nonetheless, as we argue in the main text, it should still lead to low (and positive) values of the maximal Lyapunov exponent if L is large enough. Our numerical experiments, described in detail below, show that this is already true even for $L = 2$ and $L = 3$, as long as q_L is not too close to 1.

First, we test whether our adaptation algorithm brings the activity levels q_i close to the desired values $\hat{q}_k = kq_L/L$. In two-level networks the order parameters quickly converge to the steady state, where they fluctuate around the set point (Fig. 1, top). The associated values of the control parameters feature lower level of fluctuations whose means are close to, but may not match exactly, the values predicted by the mean-field theory (Fig. 1, bottom). The fluctuations and the bias are expected to be finite-size effects. The results are similar in three-level networks (Fig. 2). Occasionally, all order and control parameters seem to converge to values slightly larger than desired. This happens because in these networks q_0, which denotes the squared mean activity of the network, converges to a significant non-zero values. Our algorithm does not attempt control q_0, since in our simple formulation of the model there is no associated control parameter and in the thermodynamic limit q_0 is predicted to be equal to 0 anyway. Given that Δ_1 is calculated as $q_1 - q_0$, the convergence of all Δ_i to $\hat{q}_L/L$ does not imply the convergence of all q_i to $i\hat{q}_L/L$. Instead, q_i converges to $i\hat{q}_L/L + q_0$. This phenomenon can lead to a failure of the algorithm when $\hat{q}_L + q_0 > 1$, which we occasionally observe in four-level networks when the number of populations is relatively small (Fig. 3). However, we expect this to be a finite-size effect that does not persist in larger networks. Indeed, increasing the number of populations in four-level networks seems to significantly reduce the effect (Fig. 4).

We repeat this procedure for various values of $\hat{q}_L$, as summarized in Fig. 5. As expected, the adaptation process drives q_L close to $\hat{q}_L$ and, in most cases, brings σ_L to values predicted by the mean-field theory. The only exception is for $L = 4$, where the σ_L resulting from the adaptation remains significantly larger than the mean-field prediction, presumably due to finite-size effects. Given that our algorithm successfully balances activity across levels, we expect it to position the system near the edge of chaos. The right panel of Fig. 5 confirms this, showing that the maximal Lyapunov exponents remain significantly closer to 0 for modular networks ($L = 2$ and $L = 3$) compared to homogeneous networks ($L = 1$). The estimated values of λ_{max} align with the mean-field predictions, although modular networks exhibit relatively large variability across realizations. Despite these finite-size effects, we conclude that balancing activity across levels in modular networks places neural dynamics in the vicinity of the edge of chaos.

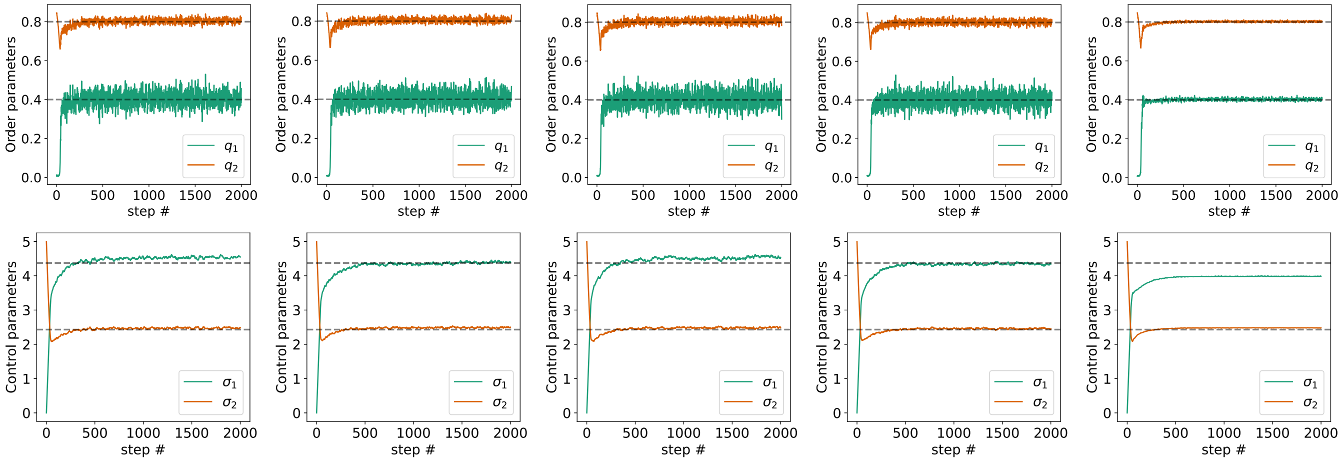

FIG. 1. Evolution of order (*top*) and control (*bottom*) parameters during the adaptation process in networks with two levels ($P_1 = P_2 = 100$). Five columns correspond to different seeds (i.e., independent realizations of the weights and initial conditions). Dashed lines denote the desired activity levels (*top*) and the corresponding control parameters, as predicted by the mean-field equations (*bottom*). Other parameters: $\eta = 0.2$, $\hat{q}_L = 0.8$.

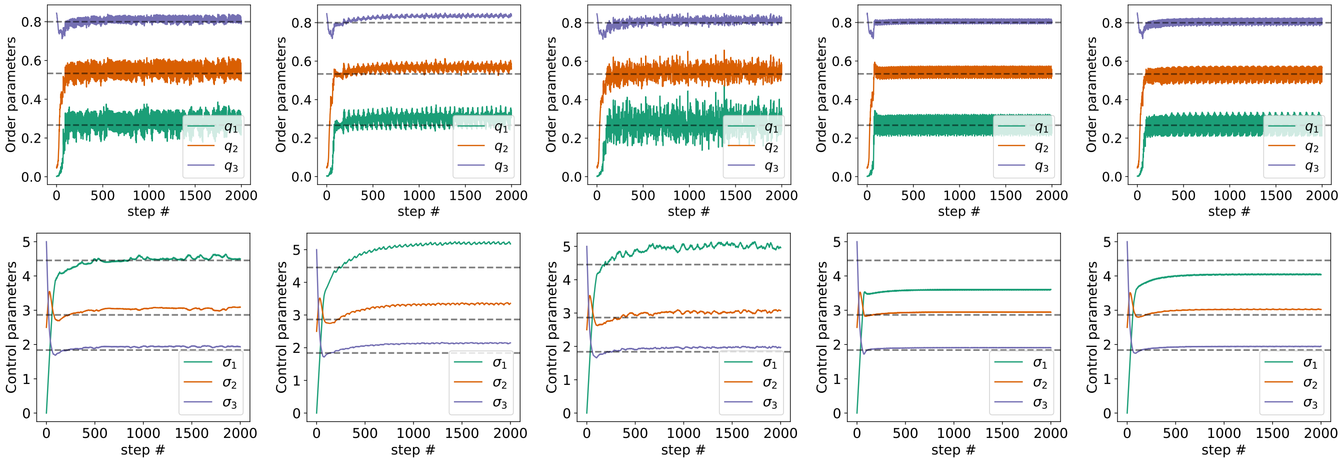

FIG. 2. Same as Fig. 1 but in networks with three levels ($P_1 = P_2 = P_3 = 22$).

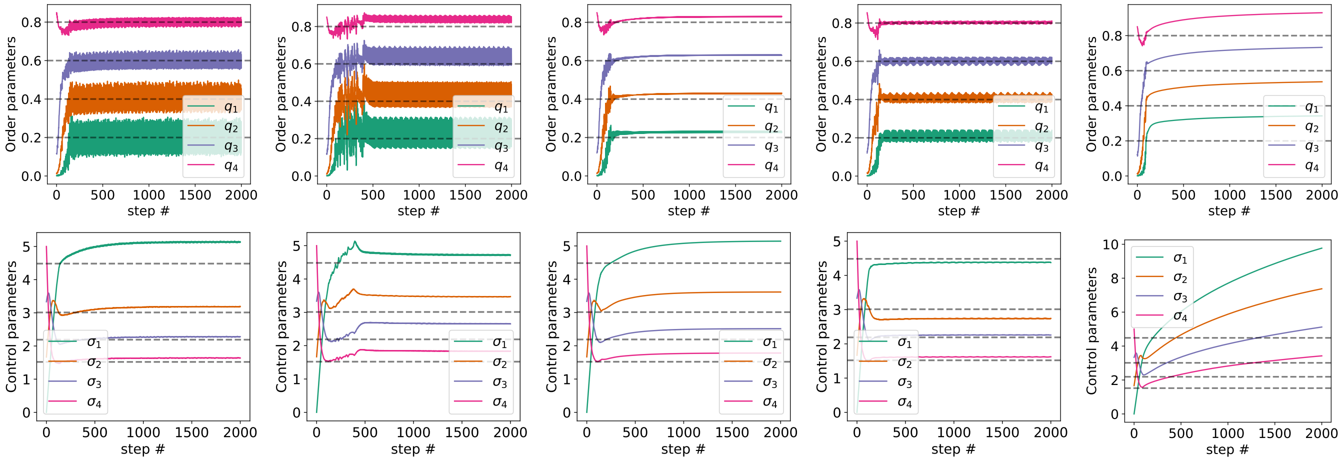

FIG. 3. Same as Fig. 1 but in networks with four levels ($P_1 = P_2 = P_3 = P_4 = 10$).

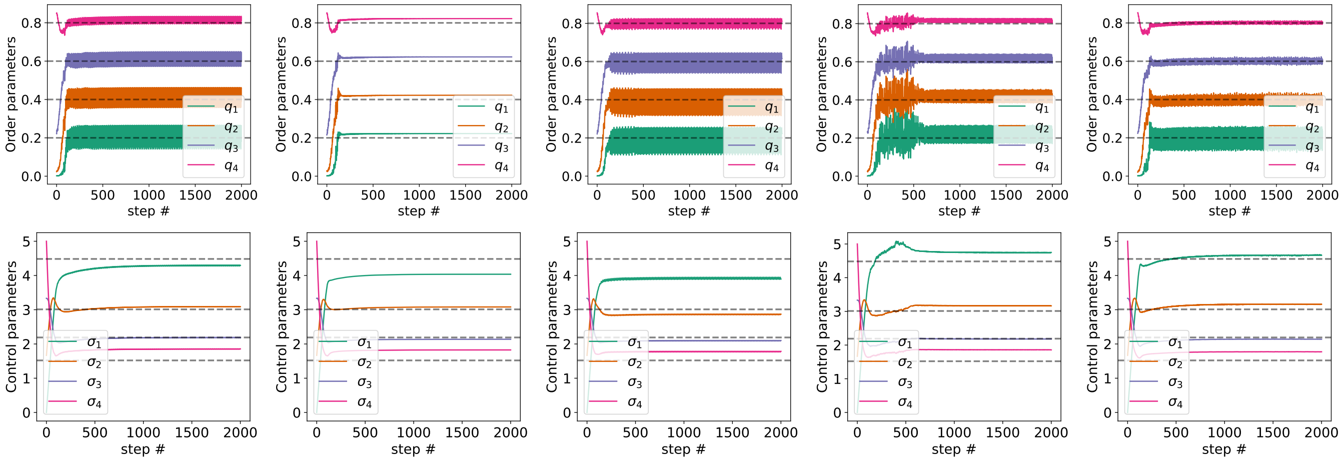

FIG. 4. Same as Fig. 3 but in larger networks with significantly more top-level populations ($P_1 = 20$, $P_2 = 15$, $P_3 = 10$, $P_4 = 5$).

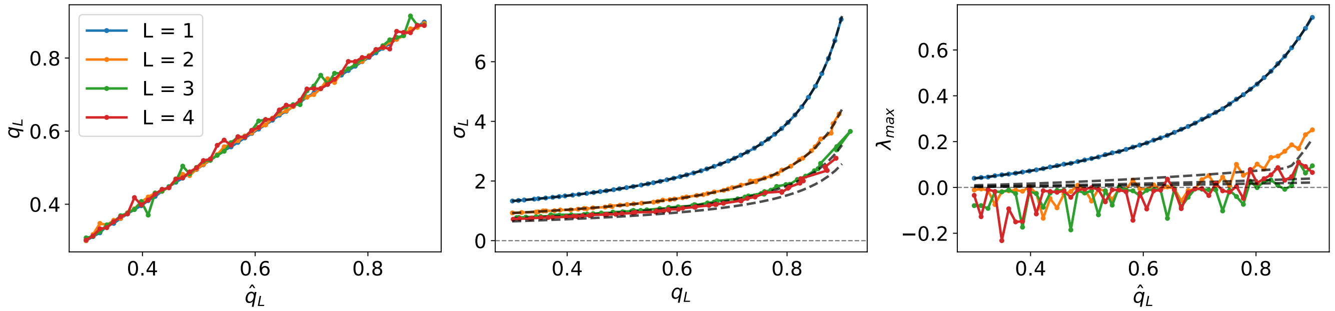

FIG. 5. Values of q_L (*left*), σ_L (*center*), and the maximal Lyapunov exponent (*right*) at the end of the adaptation process as functions of $\hat{q}_L$ or q_L for $L = 1$ ($P_1 = 10^4$), $L = 2$ ($P_1 = P_2 = 100$), $L = 3$ ($P_1 = P_2 = P_3 = 22$), and $L = 4$ ($P_1 = 20$, $P_2 = P_3 = 10$, $P_4 = 5$). The adaptation process runs for 1000 steps, after which control parameters are frozen and the simulation is restarted from a new random initial condition for 700 steps. The last 350 steps are used for estimating the order parameters q_i, whereas the last 200 steps are used for estimating λ_{max}.
